# Supplementary material for: Dittrichia graveolens (L.) Greuter, a Rapidly Spreading Invasive Plant: Chemistry and Bioactivity
Source: Molecules. 2022 Jan 28;27(3):895. doi: 10.3390/molecules27030895 (PMC8840657; doi:10.3390/molecules27030895)
Supplement: Supplementary file 1 [file molecules-27-00895-s001.zip › molecules-1497751-supplementary.pdf]

molecules

MDPI

Table S1. Bioactive metabolites identified in *Dittrichia graveolens* L.

	PHENOLIC COMPOUNDS AND DERIVATIVES			
	**Compound**	**Formula**	**Structure**	**Part of *Dittrichia graveolens* L.**
**1**	Acetophenone	C_8H_8O	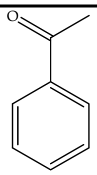	Essential oil [28, 29]
**2**	Acetophenone, 4-methyl	$C_9H_{10}O$	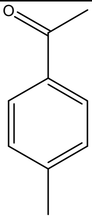	Essential oil [19]
**3**	Benzoic acid, 4-OH (syn. Benzoic acid, *p*-hydroxy)	$C_7H_6O_3$	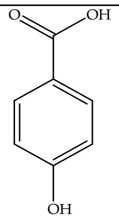	Aerial parts [17]<br>Leaves [32]
**4**	Benzoic acid derivative 1	$C_{12}H_{14}O_3$	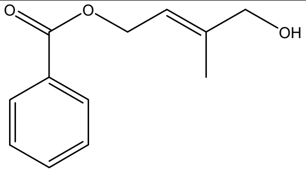	Aerial parts [15]
**5**	Benzoic acid derivative 2	$C_{12}H_{14}O_4$	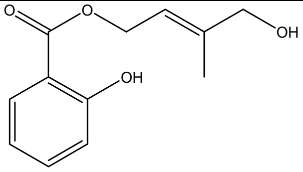	Aerial parts [15]
**6**	Benzyl salicylate	$C_{14}H_{12}O_3$	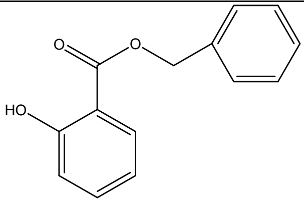	Essential oil [26]
**7**	Chlorogenic acid	$C_{16}H_{18}O_9$	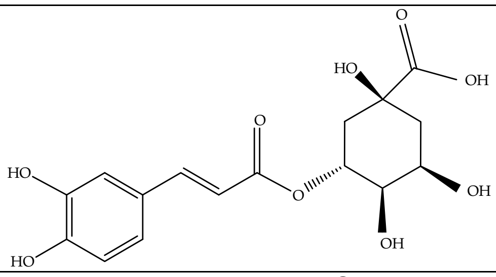	Leaves [32]
**8**	*p*-Coumaric acid	$C_9H_8O_3$	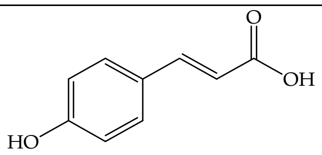	Leaves [32]

		PHENOLIC COMPOUNDS AND DERIVATIVES		
	**Compound**	**Formula**	**Structure**	**Part of *Dittrichia graveolens* L.**
**9**	Eugenol	$C_{10}H_{12}O_2$	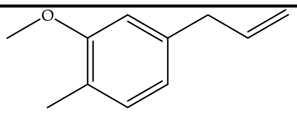	Essential oil [16]
**10**	(*E*)-*β*-Ionone	$C_{13}H_{20}O$	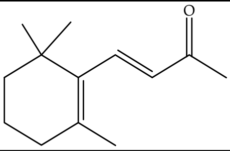	Essential oil [19, 28]
**11**	Protocatechuic acid	$C_7H_6O_4$	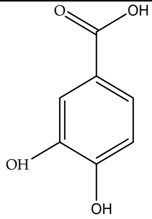	Leaves [32]
**12**	Salicylic acid	$C_7H_6O_3$	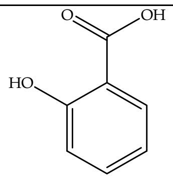	Leaves [32]
**13**	Tannic acid	$C_{76}H_{52}O_{46}$	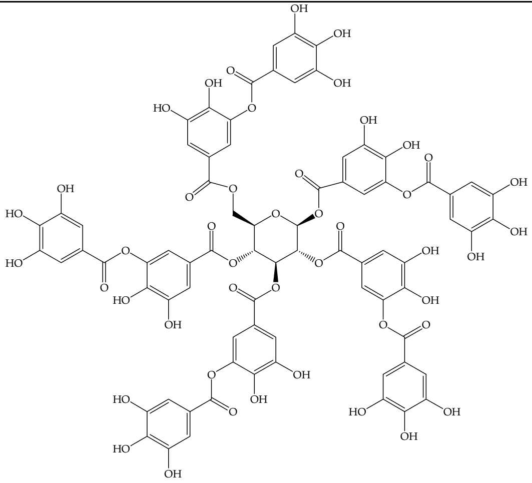	Leaves [32]
**14**	Quinic acid	$C_7H_{12}O_6$	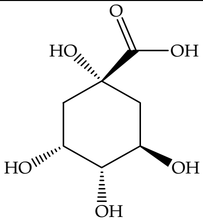	Leaves [32]

		FLAVONOIDS AND DERIVATIVES		
	**Compound**	**Formula**	**Structure**	**Part of *Dittrichia graveolens* L.**
**15**	Aromadendrin	$C_{15}H_{12}O_6$	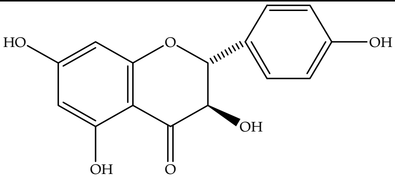	Aerial parts [17]

	**FLAVONOIDS AND DERIVATIVES**			
	**Compound**	**Formula**	**Structure**	**Part of *Dittrichia graveolens* L.**
**16**	Aromadendrin, 3-*epi*-acetyl-7-*O*-methyl	$C_{18}H_{16}O_6$	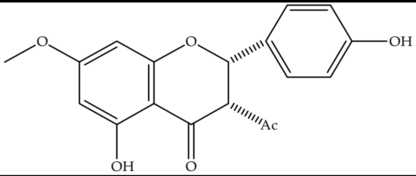	Aerial parts [17]
**17**	Aromadendrin, 3-*O*-acetyl-7-*O*-methyl	$C_{18}H_{16}O_7$	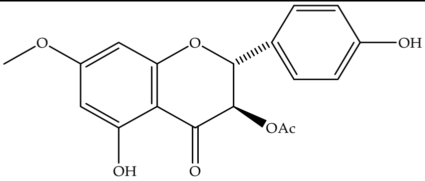	Aerial parts [17]
**18**	Aromadendrin, 7-*O*-methyl	$C_{16}H_{14}O_6$	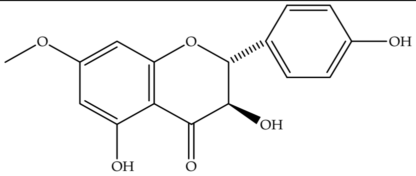	Aerial parts [17]
**19**	Catechin	$C_{15}H_{14}O_6$	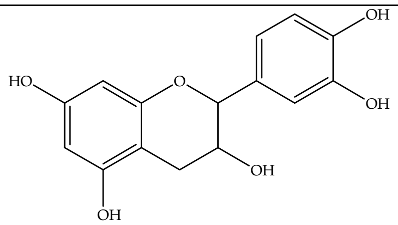	Aqueous residue of leaves and flowers hydrodistillation [11]
**20**	Hesperetin	$C_{16}H_{14}O_6$	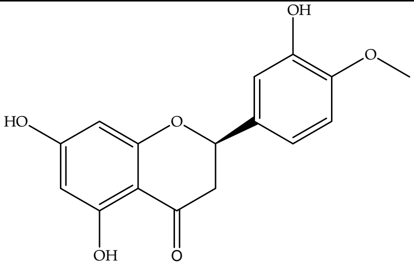	Leaves [32]
**21**	Hyperoside	$C_{21}H_{20}O_{12}$	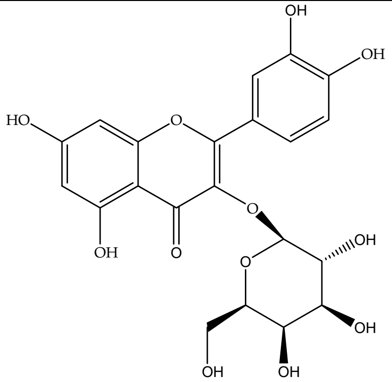	Leaves [32]
**22**	Kaempferol	$C_{15}H_{12}O_6$	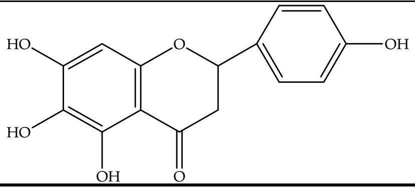	Aerial parts [17]

	Compound	Formula	Structure	Part of *Dittrichia graveolens* L.
		**FLAVONOIDS AND DERIVATIVES**		
**23**	Kaempferol, 3-methyl ether	$C_{16}H_{14}O_6$	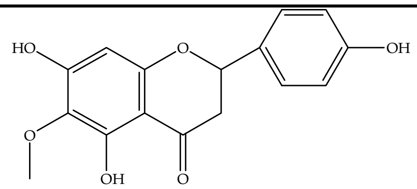	Aerial parts [17]
**24**	Kaempferol, 6-hydroxy-3,6-dimethylether	$C_{17}H_{16}O_7$	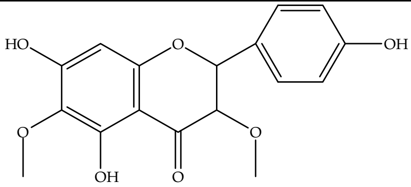	Aerial parts [17]
**25**	Padmatin	$C_{16}H_{14}O_7$	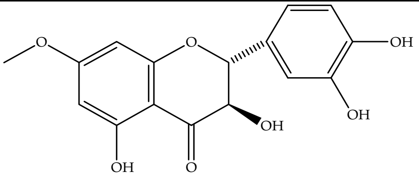	Aerial parts [17]
**26**	Padmatin, 3-*epi*	$C_{16}H_{16}O_6$	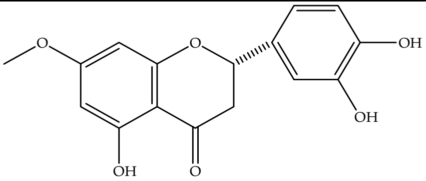	Aerial parts [17]
**27**	Padmatin, 3-*O*-acetyl	$C_{18}H_{16}O_8$	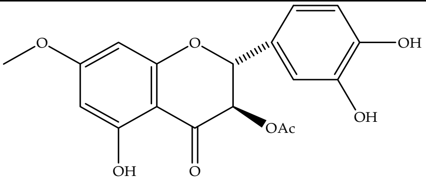	Aerial parts [17]
**28**	Quercetin	$C_{15}H_{10}O_7$	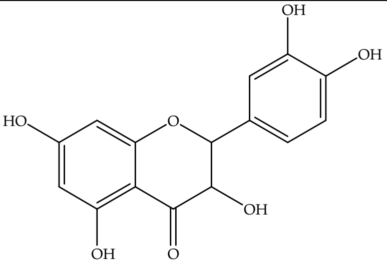	Aqueous residue of leaves and flowers hydrodistillation [11]<br><br>Leaves [32]
**29**	Sakuranetin	$C_{16}H_{14}O_5$	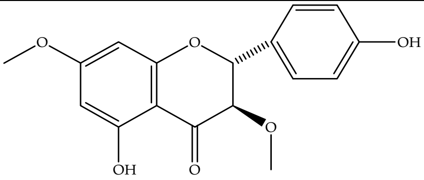	Aerial parts [17]
**30**	Scutellarein, 6-methylether	$C_{16}H_{14}O_6$	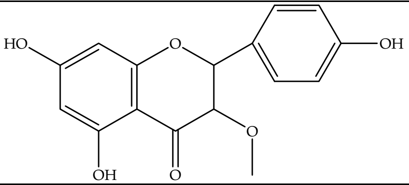	Aerial parts [17]

FLAVONOIDS AND DERIVATIVES				
	**Compound**	**Formula**	**Structure**	**Part of *Dittrichia graveolens* L.**
**31**	Taxifolin, 3-*O*-acetyl	$C_{17}H_{14}O_8$	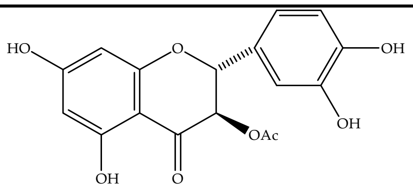	Aerial parts [17]

TERPENES AND DERIVATIVES				
	**Compound**	**Formula**	**Structure**	**Part of *Dittrichia graveolens* L.**
**32**	4-*epi* Abietol	$C_{20}H_{30}$	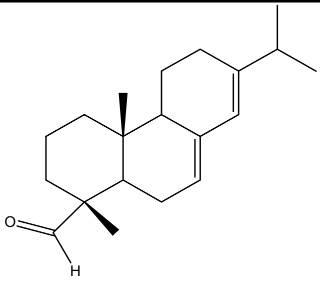	Essential oil [30]
**33**	Chrysanthenone	$C_{10}H_{14}O$	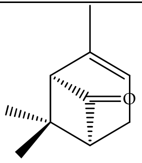	Essential oil [20]
**34**	Laurenene	$C_{20}H_{32}$	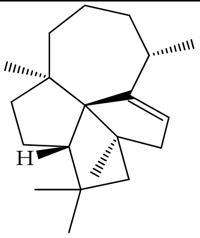	Essential oil [25]
**35**	Longipinanol	$C_{15}H_{26}O$	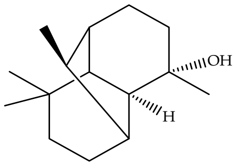	Essential oil [25]<br>Supercritical fluid extraction (SFE) extract [25]
**36**	Pinocarvone	$C_{10}H_{14}O$	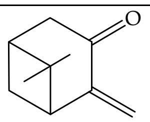	Essential oil [10]
**37**	*trans*-Pinocarveol	$C_{10}H_{16}O$	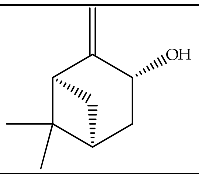	Essential oil [10, 20]

	**MONOTERPENES AND DERIVATIVES**			
	**Compound**	**Formula**	**Structure**	**Part of *Dittrichia graveolens* L.**
**38**	Artemisia alcohol	$C_{10}H_{18}O$	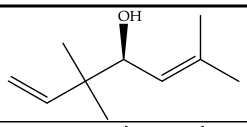	Essential oil [19, 29]
**39**	*β*-Bisabolenal	$C_{14}H_{22}O$	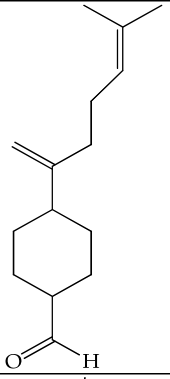	Essential oil [25]<br><br>Supercritical fluid extraction (SFE) extract [25]
**40**	Borneol	$C_{10}H_{18}O$	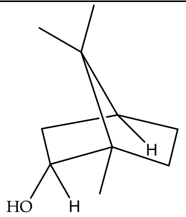	Essential oil [10, 19-24, 26-30]
**41**	Bornyl acetate	$C_{12}H_{20}O_2$	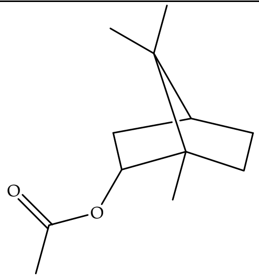	Essential oil [10, 18, 19, 21-24, 26, 28-30]
**42**	*iso* Bornyl acetate	$C_{12}H_{20}O_2$	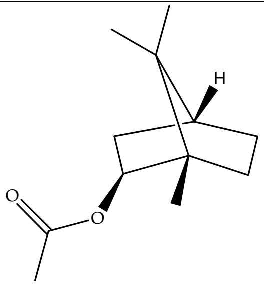	Essential oil [20]
**43**	*iso* Bornyl formate	$C_{11}H_{18}O_2$	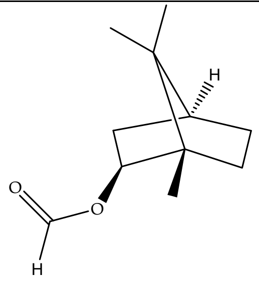	Essential oil [28]

MONOTERPENES AND DERIVATIVES				
	**Compound**	**Formula**	**Structure**	**Part of *Dittrichia graveolens* L.**
**44**	*iso* Bornyl isobutyrate, 8-isobutyryloxy	$C_{18}H_{30}O_4$	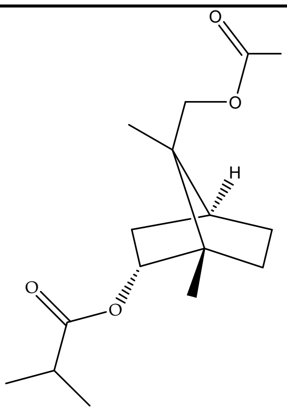	Supercritical fluid extraction (SFE) extract [25]
**45**	*iso* Bornyl isovalerate	$C_{15}H_{26}O_2$	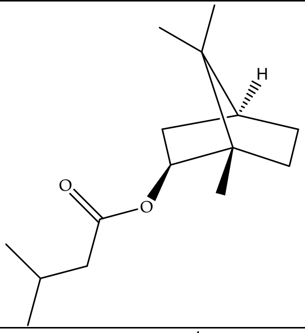	Essential oil [28]
**46**	*iso* Bornyl propanoate	$C_{13}H_{22}O_2$	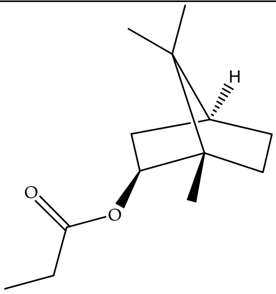	Essential oil [23, 28]
**47**	Camphene	$C_{10}H_{16}$	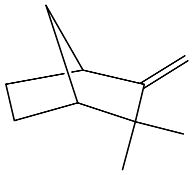	Essential oil [19-24, 28-30]
**48**	Camphene hydrate	$C_{10}H_{18}O$	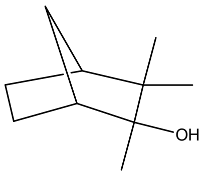	Essential oil [19, 20, 27]
**49**	Camphor	$C_{10}H_{16}O$	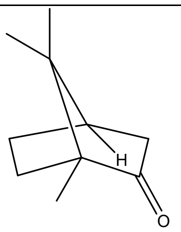	Essential oil [19, 20, 23, 27-30]
**50**	δ-2-Carene	$C_{10}H_{16}$	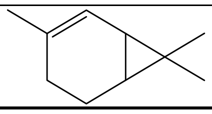	Essential oil [24]

		**MONOTERPENES AND DERIVATIVES**		
	**Compound**	**Formula**	**Structure**	**Part of *Dittrichia graveolens* L.**
**51**	Carvacrol	$C_{10}H_{14}O$	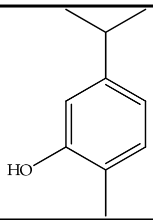	Essential oil [19]
**52**	Carveol	$C_{10}H_{16}O$	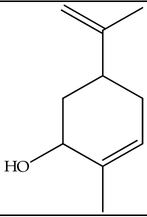	Essential oil [30]
**53**	*cis*-Carveol	$C_{10}H_{16}O$	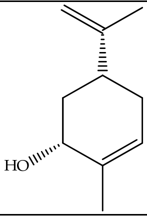	Supercritical fluid extraction (SFE) extract [25]
**54**	Carvone	$C_{10}H_{14}O$	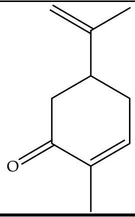	Essential oil [19]
**55**	Cineole, 1,8 (syn. Eucalyptol)	$C_{10}H_{18}O$	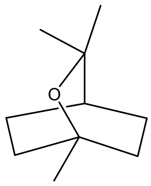	Essential oil [10, 16, 20, 21, 30]<br><br>Supercritical fluid extraction (SFE) extract [25]
**56**	Cineole, 1,8-dehydro (syn. Cineol, 2,3-dehydro-1,8)	$C_{10}H_{16}O$	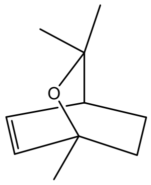	Essential oil [19, 20, 24, 28, 29]<br><br>Supercritical fluid extraction (SFE) extract [25]
**57**	*β*-Cyclocitral	$C_{10}H_{16}O$	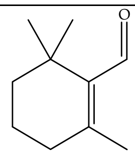	Essential oil [28]
**58**	*p*-Cymen-8-ol	$C_{10}H_{14}O$	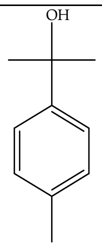	Essential oil [19, 28]

		**MONOTERPENES AND DERIVATIVES**		
	**Compound**	**Formula**	**Structure**	**Part of *Dittrichia graveolens* L.**
**59**	*p*-Cymen-9-ol	$C_{10}H_{14}O$	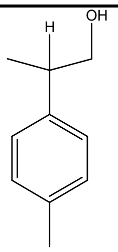	Essential oil [20]
**60**	*m*-Cymene	$C_{10}H_{14}$	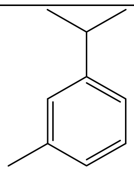	Essential oil [28]
**61**	*p*-Cymene	$C_{10}H_{14}$	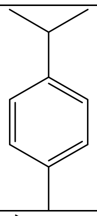	Essential oil [10, 19, 23, 28, 29]
**62**	*p*-Cymenene	$C_{10}H_{12}$	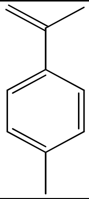	Essential oil [19, 23, 29]
**63**	(*E*)-*β*-Damascenone	$C_{13}H_{18}O$	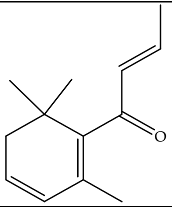	Essential oil [23, 28]
**64**	Fenchol	$C_{10}H_{18}O$	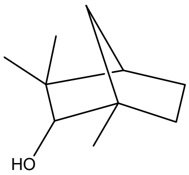	Essential oil [10, 19]
**65**	*Exo*-Fenchyl alcohol	$C_{10}H_{18}O$	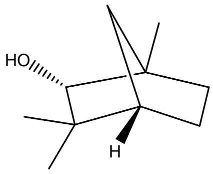	Essential oil [20]
**66**	Geranyl acetone	$C_{13}H_{22}O$	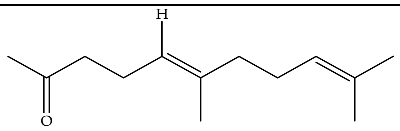	Essential oil [19, 28]
**67**	*neo*-Isothujan-3-ol	$C_{10}H_{18}O$	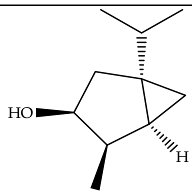	Essential oil [28]

MONOTERPENES AND DERIVATIVES				
	**Compound**	**Formula**	**Structure**	**Part of *Dittrichia graveolens* L.**
**68**	Lavandulol	$C_{10}H_{18}O$	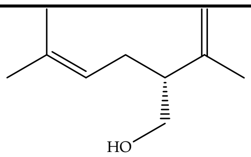	Essential oil [19]
**69**	Lavandulol acetate	$C_{12}H_{20}O_3$	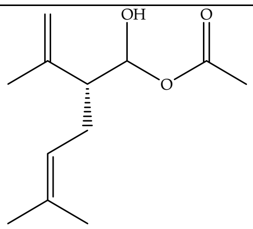	Essential oil [24]
**70**	Limonene	$C_{10}H_{16}$	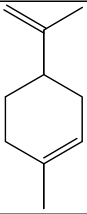	Essential oil [19, 22, 23, 28, 29]
**71**	L-Limonene	$C_{10}H_{16}$	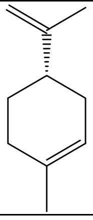	Essential oil [20, 30]
**72**	Linalool	$C_{10}H_{18}O$	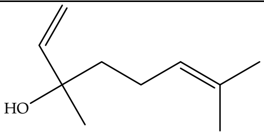	Essential oil [10, 19, 23, 29, 30]
**73**	*cis* Linalool oxide	$C_{10}H_{18}O_2$	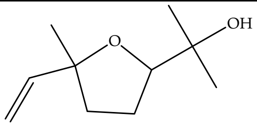	Essential oil [28]
**74**	*cis*-Linalool oxide, dehydroxy	$C_{10}H_{16}O$	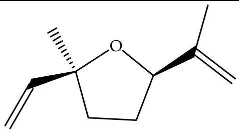	Essential oil [28]
**75**	*p*-Menth-2-en-1-ol	$C_{10}H_{18}O$	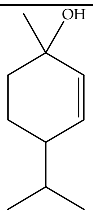	Essential oil [20]
**76**	*p*-Mentha-1(7),2-dien-8-ol (*β*-phellandren-8-ol)	$C_{10}H_{16}O$	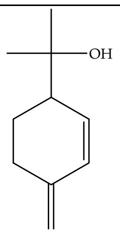	Essential oil [19]

	**MONOTERPENES AND DERIVATIVES**			
	**Compound**	**Formula**	**Structure**	**Part of *Dittrichia graveolens* L.**
**77**	*trans*-*p*-Mentha-1(7),8-dien-2-ol	$C_{10}H_{16}O$	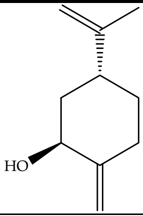	Essential oil [28]
**78**	*p*-Mentha-1,5-dien-8-ol (syn. *α*-Phellandren-8-ol)	$C_{10}H_{16}O$	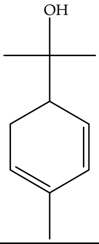	Essential oil [19, 20]
**79**	Myrcene	$C_{10}H_{16}$	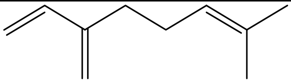	Essential oil [10, 19, 26]
**80**	Myrtenal	$C_{10}H_{14}O$	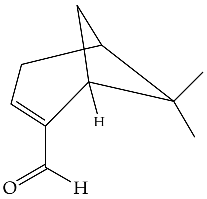	Essential oil [28]
**81**	Myrtenol	$C_{10}H_{16}O$	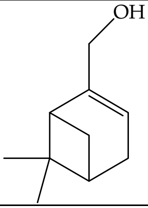	Essential oil [10]
**82**	Nerol oxide	$C_{10}H_{16}O$	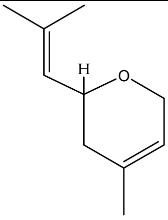	Essential oil [28, 29]
**83**	Perilla aldehyde	$C_{10}H_{14}O$	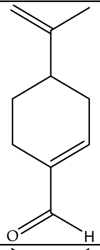	Essential oil [19, 28]
**84**	*α*-Phellandrene	$C_{10}H_{16}$	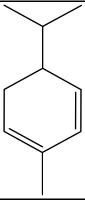	Essential oil [10, 19, 30]

	**MONOTERPENES AND DERIVATIVES**			
	**Compound**	**Formula**	**Structure**	**Part of *Dittrichia graveolens* L.**
**85**	*β*-Phellandrene	$C_{10}H_{16}$	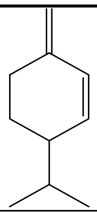	Essential oil [19]
**86**	*α*-Pinene	$C_{10}H_{16}$	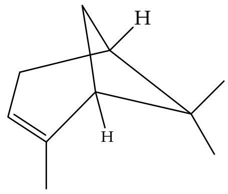	Essential oil [10, 19, 22-24, 26-30]
**87**	*β*-Pinene	$C_{10}H_{16}$	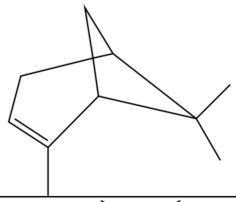	Essential oil [10, 19-24, 28-30]
**88**	Piperitone	$C_{10}H_{16}O$	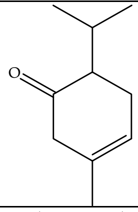	Essential oil [19, 28]
**89**	Sabinene	$C_{10}H_{16}$	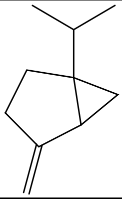	Essential oil [26]
**90**	Santolina alcohol	$C_{10}H_{18}O$	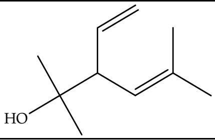	Essential oil [19, 29]
**91**	Santolinatriene	$C_{10}H_{16}$	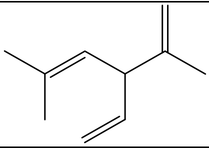	Essential oil [19, 29]
**92**	Terpinen-4-ol (syn. Terpin-4-ol)	$C_{10}H_{18}O$	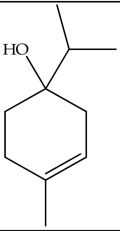	Essential oil [10, 19, 20]
**93**	*α*-Terpinene	$C_{10}H_{16}$	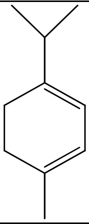	Essential oil [19, 29]

			**MONOTERPENES AND DERIVATIVES**	
	**Compound**	**Formula**	**Structure**	**Part of *Dittrichia graveolens* L.**
**94**	γ-Terpinene	$C_{10}H_{16}$	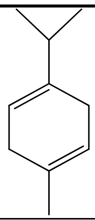	Essential oil [10, 19, 23, 25, 29]
**95**	α-Terpineol	$C_{10}H_{18}O$	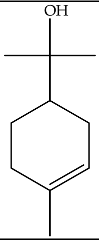	Essential oil [10, 16, 19, 21, 22, 28, 30]
**96**	Terpinolene	$C_{10}H_{16}$	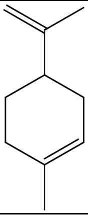	Essential oil [19, 23, 29]
**97**	*trans*-Thujone (syn. α-Thujone)	$C_{10}H_{16}O$	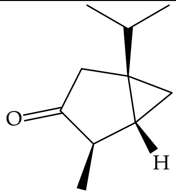	Essential oil [20]
**98**	β-Thujone	$C_{10}H_{16}O$	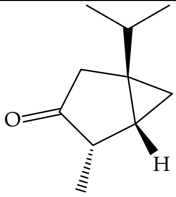	Essential oil [29, 30]
**99**	Thymol	$C_{10}H_{14}O$	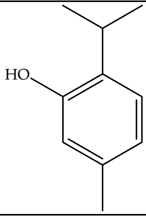	Essential oil [19, 24, 28, 30]
**100**	Thymol, methyl ether	$C_{11}H_{16}O$	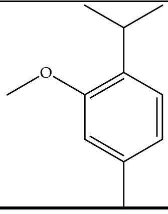	Essential oil [20, 23]

MONOTERPENES AND DERIVATIVES

	Compound	Formula	Structure	Part of *Dittrichia graveolens* L.
**101**	Vestitenone	$C_{12}H_{18}O$	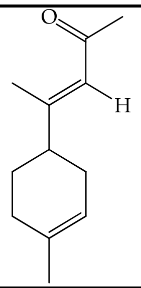	Essential oil [25]
**102**	*trans*-Vetrocitral C	$C_9H_{14}O$	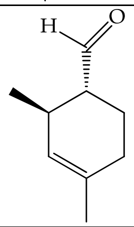	Supercritical fluid extraction (SFE) extract [25]

SESQUITERPENES

	Compound	Formula	Structure	Part of *Dittrichia graveolens* L.
**103**	1H-Cycloprop[e]azulene, 1a,2,3,4,4a,5,6, 7*β*-octahydro-1,1,4,7-tetramethyl	$C_{15}H_{24}$	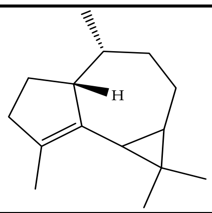	Essential oil [20]
**104**	4,5-di-*epi*-Aristolochene	$C_{15}H_{24}$	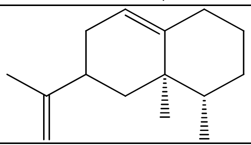	Essential oil [19, 20, 28]
**105**	Aromadendrene	$C_{15}H_{24}$	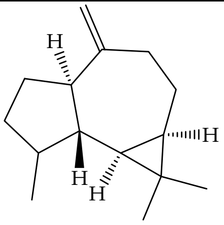	Essential oil [20, 26]
**106**	*allo*-Aromadendrene	$C_{15}H_{24}$	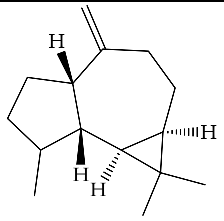	Essential oil [19, 20, 26, 27, 29]
**107**	Bigelovin	$C_{18}H_{22}O_4$	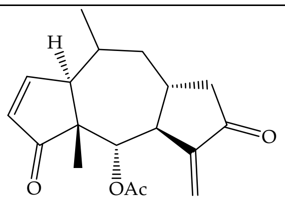	Epigeal parts extracts [13]

	**SESQUITERPENES**			
	**Compound**	**Formula**	**Structure**	**Part of *Dittrichia graveolens* L.**
**108**	α-Bisabolol	$C_{15}H_{26}O$	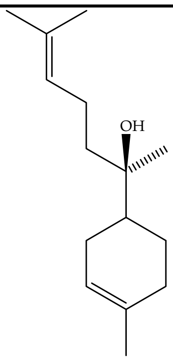	Essential oil [28]
**109**	Cadalene	$C_{15}H_{18}$	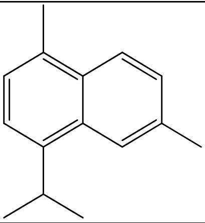	Essential oil [19]
**110**	Cadina-1,4-diene	$C_{15}H_{24}$	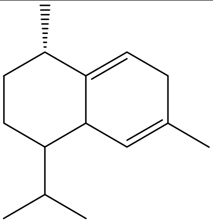	Essential oil [19]
**111**	α-Cadinene	$C_{15}H_{24}$	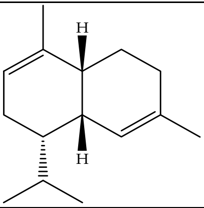	Essential oil [19, 21, 29]
**112**	γ-Cadinene	$C_{15}H_{24}$	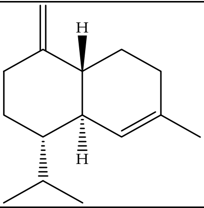	Essential oil [19, 22, 23, 29]
**113**	δ-Cadinene	$C_{15}H_{24}$	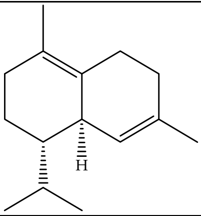	Essential oil [19, 20, 24, 26, 29, 30]
**114**	α-Cadinol	$C_{15}H_{26}O$	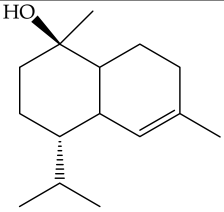	Essential oil [19]

	SESQUITERPENES			
	**Compound**	**Formula**	**Structure**	**Part of *Dittrichia graveolens* L.**
**115**	*δ*-Cadinol	$C_{15}H_{26}O$	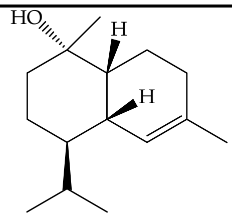	Essential oil [24]
**116**	*τ*-Cadinol (syn. *epi*-*α*-Cadinol)	$C_{15}H_{26}O$	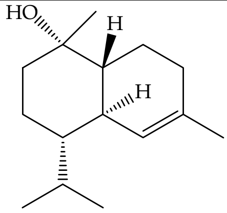	Essential oil [19, 20, 23, 26, 27, 29, 30]
**117**	*α*-Calacorene	$C_{15}H_{20}$	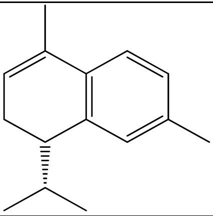	Essential oil [19]
**118**	*β*-Calacorene	$C_{15}H_{20}$	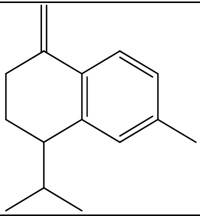	Essential oil [19]
**119**	*cis*-Calamenene	$C_{15}H_{22}$	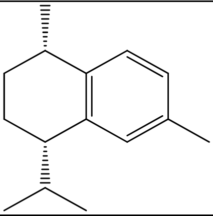	Essential oil [19]
**120**	*trans*-Calamenene	$C_{15}H_{22}$	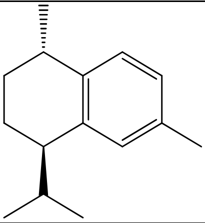	Essential oil [19]
**121**	Camphor, juniper	$C_{15}H_{26}O$	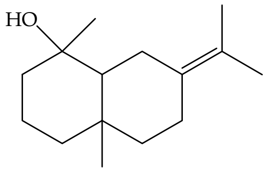	Essential oil [25]<br>Supercritical fluid extraction (SFE) extract [25]
**122**	Carabrone	$C_{15}H_{20}O_3$	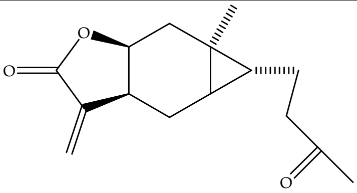	Aerial parts [17]

			**SESQUITERPENES**	
	**Compound**	**Formula**	**Structure**	**Part of *Dittrichia graveolens* L.**
**123**	Carabrone, 4H	$C_{15}H_{22}O_3$	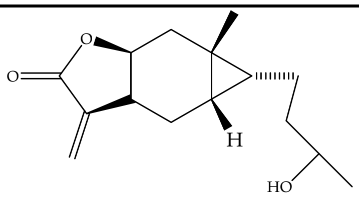	Aerial parts [17]
**124**	Caryophylla-4(12),8(13)-dien-5α-ol	$C_{15}H_{24}O$	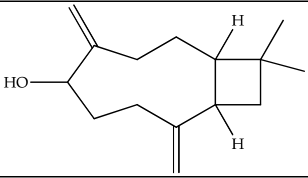	Essential oil [20]
**125**	Caryophylla-4(14),8(15)-dien-5α-ol	$C_{15}H_{26}O$	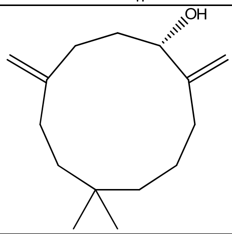	Essential oil [19]
**126**	Caryophyllene alcohol (syn. Apollanol)	$C_{15}H_{26}O$	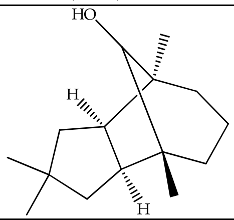	Essential oil [19]
**127**	Caryophyllene epoxide	$C_{15}H_{24}O$	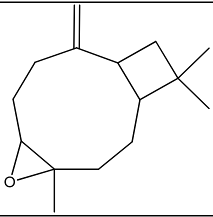	Essential oil [19]
**128**	Caryophyllene oxide	$C_{15}H_{24}O$	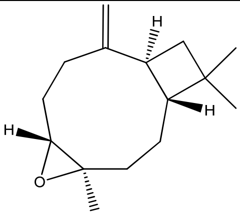	Essential oil [20, 21, 23-30]<br><br>Supercritical fluid extraction (SFE) extract [25]
**129**	*trans*-Caryophyllene (syn. β-Caryophyllene, (*E*)-Caryophyllene)	$C_{15}H_{24}$	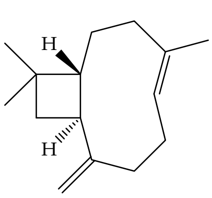	Essential oil [10, 19, 20, 22-24, 27-30]<br><br>Supercritical fluid extraction (SFE) extract [25]
**130**	γ-Caryophyllene (syn. *iso* Caryophyllene)	$C_{15}H_{24}$	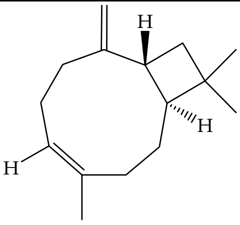	Essential oil [19, 29]

			**SESQUITERPENES**	
	**Compound**	**Formula**	**Structure**	**Part of *Dittrichia graveolens* L.**
**131**	9-*epi*-*β*-Caryophyllene, 14-hydroxy	$C_{15}H_{24}O$	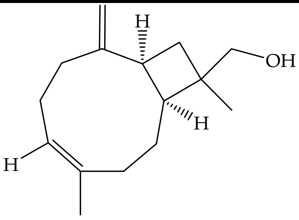	Essential oil [29]
**132**	9-*epi*-(*E*)-Caryophyllene	$C_{15}H_{24}$	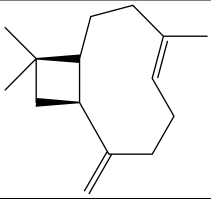	Essential oil [25]<br><br>Supercritical fluid extraction (SFE) extract [25]
**133**	Clovene	$C_{15}H_{24}$	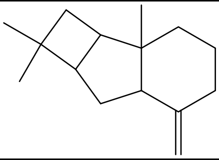	Essential oil [19]
**134**	Cedr-8(15)-en-9-*α*-ol	$C_{15}H_{24}O$	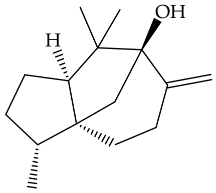	Essential oil [23, 25]<br><br>Supercritical fluid extraction (SFE) extract [25]
**135**	*α*-Copaene	$C_{15}H_{24}$	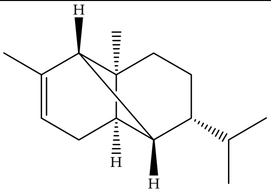	Essential oil [19]
**136**	Costic acid	$C_{15}H_{22}O_2$	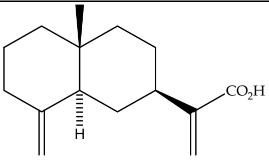	Aerial parts [17]<br><br>Epigeal parts extracts [13]
**137**	Costic acid, 10-isobutyryloxy-8,9-epoxythymol isobutyrate	$C_{15}H_{22}O_4$	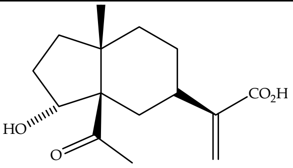	Aerial parts [15]<br><br>Essential oil [19]
**138**	Costic acid, 1*β*-hydroxy	$C_{15}H_{22}O_3$	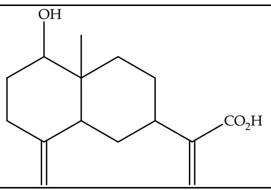	Aerial parts [17]
**139**	Costic acid, 2*α*-hydroxy	$C_{15}H_{22}O_3$	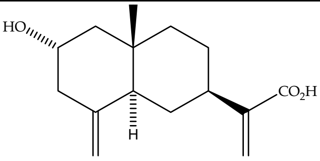	Aerial parts [17]<br><br>Epigeal parts extracts [13]

	**SESQUITERPENES**			
	**Compound**	**Formula**	**Structure**	**Part of *Dittrichia graveolens* L.**
**140**	Costic acid, 2α-hydroxy-3,4-dehydro-4,15-dihydro	$C_{15}H_{22}O_3$	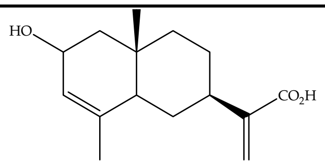	Leaves [14]
**141**	Costic acid, 3α-hydroxy (syn. Viscic acid)	$C_{15}H_{22}O_3$	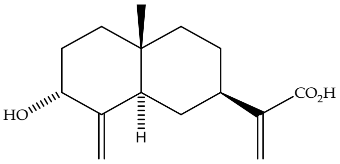	Aerial parts [17]<br><br>Epigeal parts extracts [13]<br><br>Leaves [14]
**142**	*iso* Costic acid	$C_{15}H_{22}O_2$	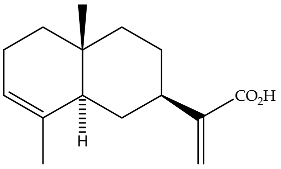	Aerial parts [17]<br><br>Epigeal parts extracts [13]<br><br>Essential oil [19]
**143**	β-Cubebene	$C_{15}H_{24}$	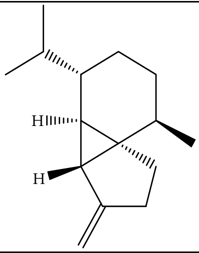	Essential oil [24]
**144**	1,10-di-*epi* Cubenol	$C_{15}H_{26}O$	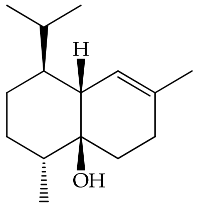	Essential oil [23, 25]<br><br>Supercritical fluid extraction (SFE) extract [25]
**145**	α-Cyperone	$C_{15}H_{22}O$	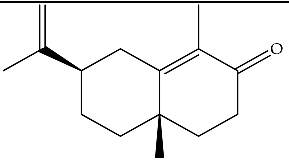	Essential oil [19, 30]
**146**	Diketone	$C_{15}H_{20}O_4$	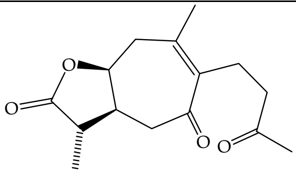	Aerial parts [15]
**147**	Eudesm, 3α-hydroxy-4-en-12,6β-olide	$C_{15}H_{20}O_3$	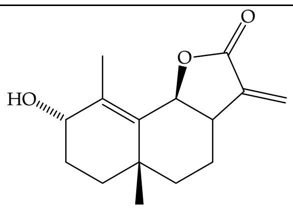	Aerial parts [17]

			**SESQUITERPENES**	
	**Compound**	**Formula**	**Structure**	**Part of *Dittrichia graveolens* L.**
**148**	Eudesma, 12-carboxy,3,11(13)-diene	$C_{15}H_{22}O_2$	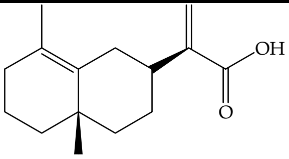	Leaves [14]
**149**	6-Eudesmen-4-α-ol	$C_{15}H_{26}O$	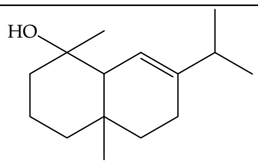	Essential oil [19]
**150**	α-Eudesmol	$C_{15}H_{26}O$	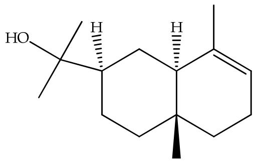	Essential oil [25]<br>Supercritical fluid extraction (SFE) extract [25]
**151**	β-Eudesmol	$C_{15}H_{26}O$	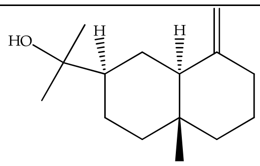	Essential oil [19]
**152**	(*E*)-β-Farnesene	$C_{15}H_{24}$	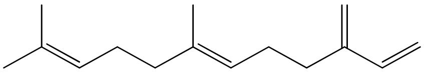	Essential oil [28]
**153**	(*E*,*E*)-α-Farnesene	$C_{15}H_{24}$	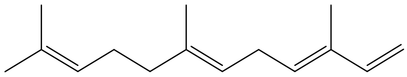	Essential oil [25]<br>Supercritical fluid extraction (SFE) extract [25]
**154**	β-*trans*-Farnesene	$C_{15}H_{24}$	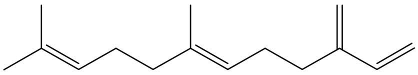	Essential oil [26]
**155**	(2*E*,6*E*)-Farnesol	$C_{15}H_{26}O$	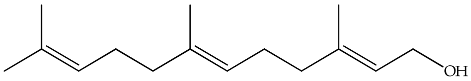	Essential oil [29, 30]
**156**	Germacrene-D	$C_{15}H_{24}$	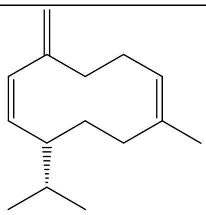	Essential oil [20, 29]

			SESQUITERPENES	
	**Compound**	**Formula**	**Structure**	**Part of *Dittrichia graveolens* L.**
**157**	Globulol	$C_{15}H_{26}O$	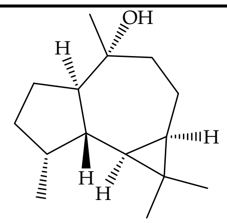	Essential oil [25]<br><br>Supercritical fluid extraction (SFE) extract [25]
**158**	Graveolide	$C_{15}H_{20}O_3$	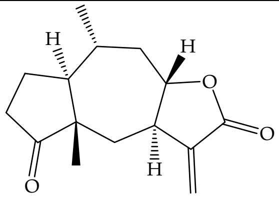	Epigeal parts extracts [13]
**159**	*trans*-*β*-Guaiene	$C_{15}H_{24}$	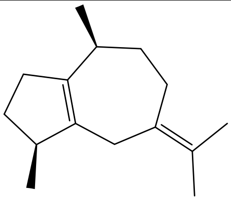	Essential oil [25]<br><br>Supercritical fluid extraction (SFE) extract [25]
**160**	*α*-Guaiene	$C_{15}H_{24}$	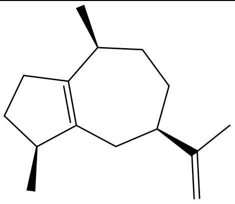	Essential oil [23, 29]
**161**	Guaiol acetate	$C_{17}H_{28}O_2$	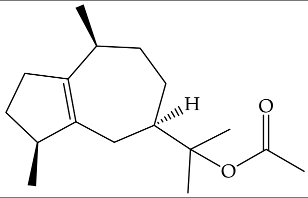	Essential oil [25]<br><br>Supercritical fluid extraction (SFE) extract [25]
**162**	*α*-Gurjunene	$C_{15}H_{24}$	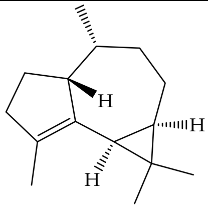	Essential oil [29]
**163**	*γ*-Gurjunene	$C_{15}H_{24}$	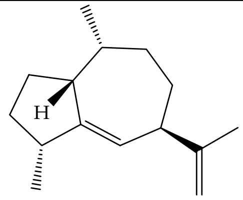	Essential oil [25]<br><br>Supercritical fluid extraction (SFE) extract [25]
**164**	8-*epi*-Helenalin	$C_{15}H_{18}O_4$	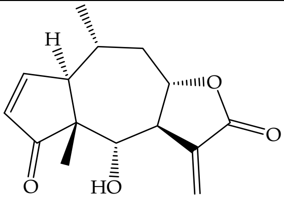	Epigeal parts extracts [13]

			**SESQUITERPENES**	
	**Compound**	**Formula**	**Structure**	**Part of *Dittrichia graveolens* L.**
**165**	γ-Himachalene	$C_{15}H_{24}$	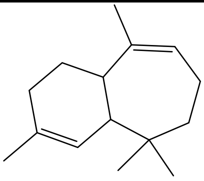	Essential oil [25]<br><br>Supercritical fluid extraction (SFE) extract [25]
**166**	Himachalol	$C_{15}H_{26}O$	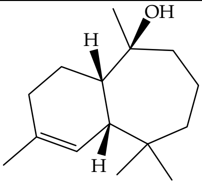	Essential oil [25]<br><br>Supercritical fluid extraction (SFE) extract [25]
**167**	Humulene	$C_{15}H_{24}$	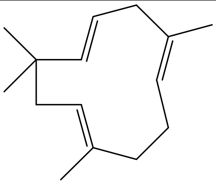	Essential oil [24]
**168**	α-Humulene	$C_{15}H_{24}$	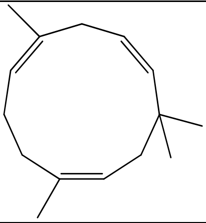	Essential oil [10, 19, 23, 24, 27-30]
**169**	Ilicic acid	$C_{15}H_{24}O_3$	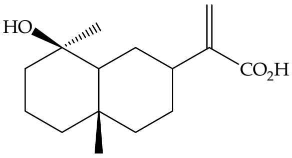	Aerial parts [17]<br><br>Epigeal parts extracts [13]<br><br>Leaves [14]
**170**	Ilicic acid methyl ester (syn. Vachanic acid)	$C_{16}H_{26}O_3$	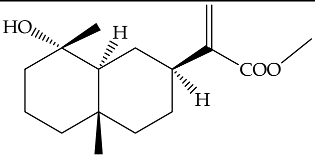	Epigeal parts extracts [13]
**171**	Ilicic acid methyl ester, 3α-hydroxy	$C_{16}H_{26}O_4$	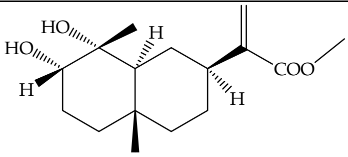	Epigeal parts extracts [13]
**172**	Ilicic acid methyl ester, 3α-acetyl	$C_{18}H_{28}O_5$	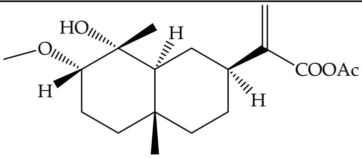	Epigeal parts extracts [13]
**173**	Ilicic acid, 2α-hydroxy-4-*epi*	$C_{15}H_{24}O_4$	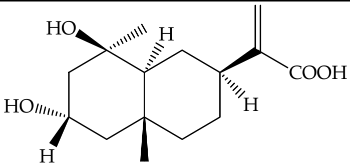	Epigeal parts extracts [13]

			**SESQUITERPENES**	
	**Compound**	**Formula**	**Structure**	**Part of *Dittrichia graveolens* L.**
**174**	Ilicic acid, 3α-hydroxy	$C_{15}H_{24}O_4$	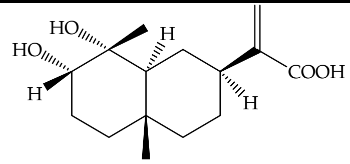	Epigeal parts extracts [13]
**175**	Ilicic acid, 3α-hydroxy-4-*epi*	$C_{14}H_{22}O_4$	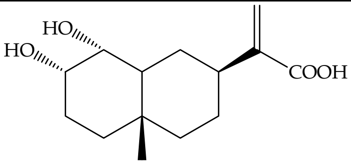	Epigeal parts extracts [13]
**176**	*neo* Intermedeol	$C_{15}H_{26}O$	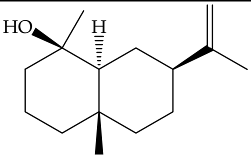	Essential oil [20, 30]
**177**	Ivalin	$C_{15}H_{20}O_3$	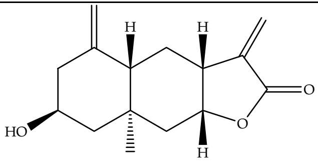	Aerial parts [34]
**178**	Ivalin, acetate (syn. Acetylivalin)	$C_{17}H_{22}O_4$	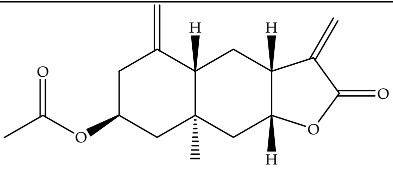	Aerial parts [34]
**179**	Manool	$C_{20}H_{34}O$	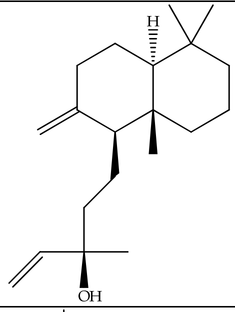	Essential oil [30]
**180**	α-Muurolene	$C_{15}H_{24}$	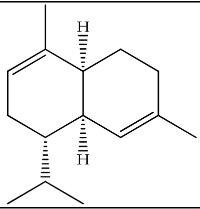	Essential oil [10, 19, 23, 29]
**181**	γ-Muurolene	$C_{15}H_{24}$	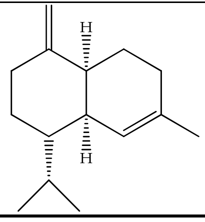	Essential oil [19, 23, 30]

			**SESQUITERPENES**	
	**Compound**	**Formula**	**Structure**	**Part of *Dittrichia graveolens* L.**
**182**	τ-Muurolol (syn. *epi*-α-Muurolol)	$C_{15}H_{26}O$	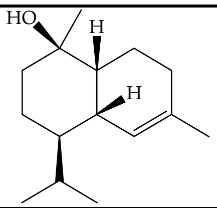	Essential oil [19, 29, 30]
**183**	Oplopanone	$C_{15}H_{26}O_2$	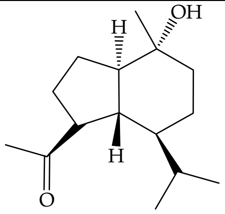	Supercritical fluid extraction (SFE) extract [25]
**184**	Presilphiperfol-7-ene	$C_{15}H_{24}$	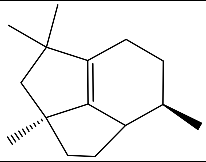	Essential oil [19]
**185**	Selin-11-en-4-*α*-ol	$C_{15}H_{26}O$	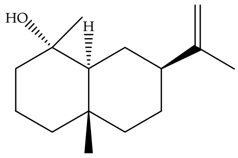	Essential oil [19, 23, 25, 28]<br><br>Supercritical fluid extraction (SFE) extract [25]
**186**	*β*-Selinene	$C_{15}H_{24}$	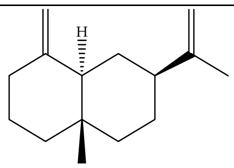	Essential oil [19, 27, 28, 30]
**187**	(*E*)-Sesquilavandulol	$C_{15}H_{26}O$	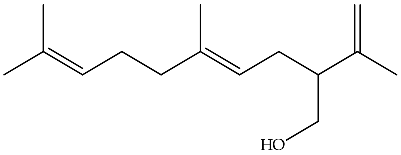	Essential oil [25]<br><br>Supercritical fluid extraction (SFE) extract [25]
**188**	(*E*)-Sesquilavandulyl acetate	$C_{17}H_{28}O_2$	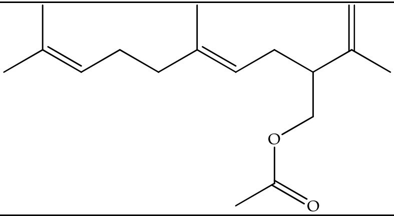	Essential oil [28]
**189**	Tomentosin (syn. Xanthalongin)	$C_{15}H_{20}O_3$	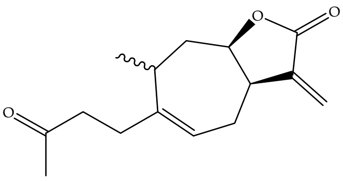	Aerial parts [17]<br><br>Epigeal parts extracts [13]<br><br>Leaves [14]

	**SESQUITERPENES**			
	**Compound**	**Formula**	**Structure**	**Part of *Dittrichia graveolens* L.**
**190**	8-*epi* Tomentosin	$C_{15}H_{20}O_3$	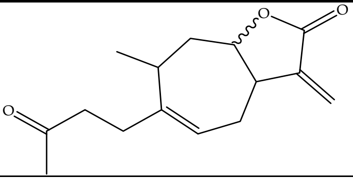	Aerial parts [15]
**191**	10-*epi* Tomentosin	$C_{15}H_{20}O_3$	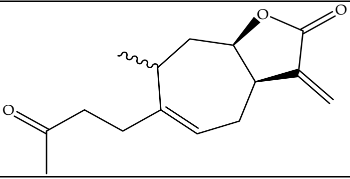	Leaves [14]
**192**	Tomentosin derivative	$C_{17}H_{26}O_4$	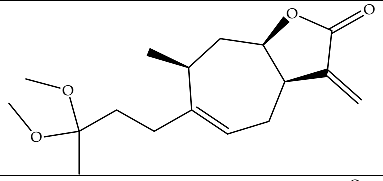	Leaves [14]
**193**	Tomentosin, 11α,13-dihydro derivative	$C_{15}H_{22}O_3$	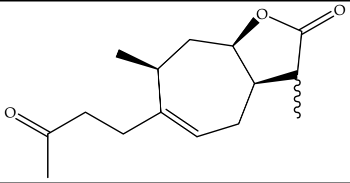	Leaves [14]
**194**	Tomentosin, 11β,13-dihydro derivative	$C_{15}H_{22}O_3$	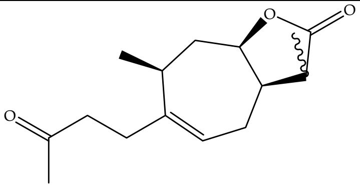	Leaves [14]
**195**	Tomentosin, 4H	$C_{15}H_{22}O_3$	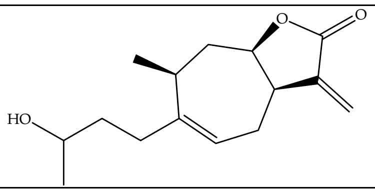	Aerial parts [17]<br><br>Epigeal parts extracts [13]
**196**	Valencene	$C_{15}H_{24}$	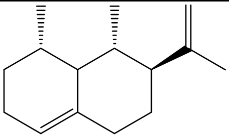	Essential oil [23]
**197**	Viridiflorol	$C_{15}H_{26}O$	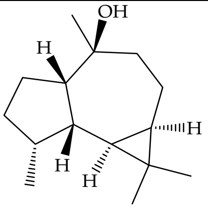	Essential oil [25]<br><br>Supercritical fluid extraction (SFE) extract [25]
**198**	Xanthalongin, 2α-hydroxy-2*R*	$C_{15}H_{20}O_4$	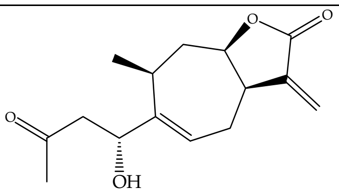	Epigeal parts extracts [13]

SESQUITERPENES				
	**Compound**	**Formula**	**Structure**	**Part of *Dittrichia graveolens* L.**
**199**	Xanthatin,8-*epi*-l*β*,5*β*-epoxide, 11,13-dihydro derivative	$C_{15}H_{18}O_4$	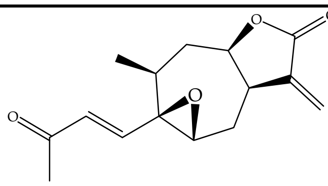	Leaves [14]
**200**	Zonarene	$C_{15}H_{24}$	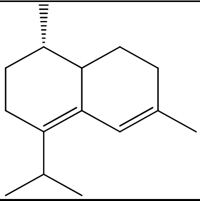	Essential oil [19]

GUAIANOLIDES AND PSEUDOGUAIANOLIDES				
	**Compound**	**Formula**	**Structure**	**Part of *Dittrichia graveolens* L.**
**201**	3,4*α*,8-trimethyl dodecahydro azuleno[6,5-*β*]furan-2,5-dione	$C_{15}H_{22}O_3$	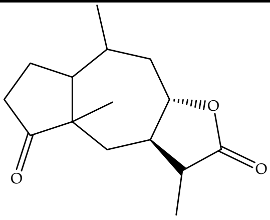	Aerial parts [35]
**202**	Carboxylic acid, 7,7-dimethyl-6-methylidenetricyclo[6.2.1.0^{1,5}]undecane-2	$C_{15}H_{22}O_2$	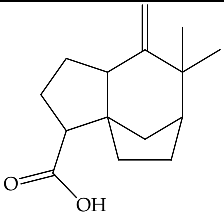	aerial parts [36]
**203**	Confertin	$C_{16}H_{24}O_4$	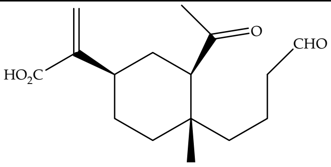	Aerial parts [15]
**204**	Inuviscolide	$C_{15}H_{20}O_3$	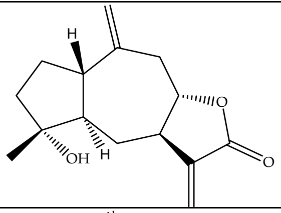	Leaves [14]
**205**	Inuviscolide, 11*β*,13-dihydro	$C_{15}H_{22}O_3$	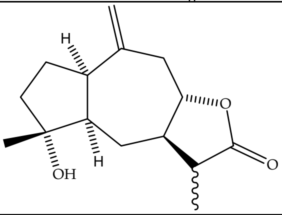	Aerial parts [15]

GUAIANOLIDES AND PSEUDOGUAIANOLIDES				
	**Compound**	**Formula**	**Structure**	**Part of *Dittrichia graveolens* L.**
**206**	4-*epi*-*iso* Inuviscolide	$C_{15}H_{20}O_3$	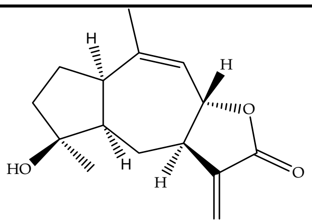	Epigeal parts extracts [13]
**207**	Inuviscolide, 4*α*,5*α*-epoxy	$C_{15}H_{20}O_3$	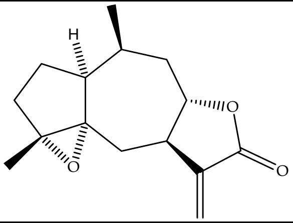	Aerial parts [17]
**208**	Inuviscolide, l-epimer	$C_{15}H_{20}O_3$	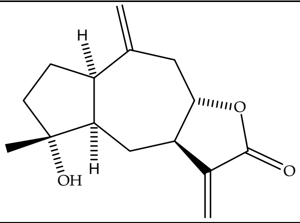	Aerial parts [15]
**209**	Inuviscotide, 11*β*,13-dihydro	$C_{15}H_{22}O_3$	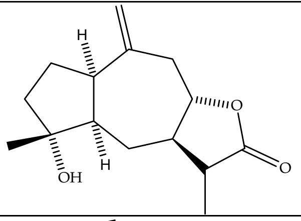	Aerial parts [15]
**210**	Pseudoguaianolide 4	$C_{15}H_{22}O_3$	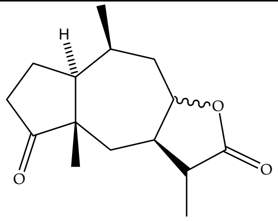	Aerial parts [15]
**211**	Pseudoguaianolide 5	$C_{15}H_{22}O_3$	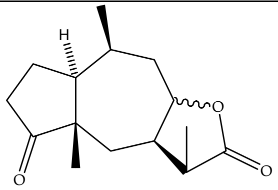	Aerial parts [15]

ORGANIC COMPOUNDS				
	**Compound**	**Formula**	**Structure**	**Part of *Dittrichia graveolens* L.**
**212**	Benzaldehyde	C_7H_6O	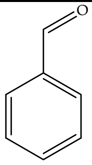	Essential oil [28, 29]

	ORGANIC COMPOUNDS			
	**Compound**	**Formula**	**Structure**	**Part of *Dittrichia graveolens* L.**
**213**	Benzene acetaldehyde	C_8H_8O	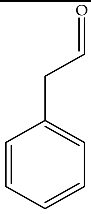	Essential oil [28]
**214**	Benzoate, 2-methylbutyl	$C_{12}H_{16}O_2$	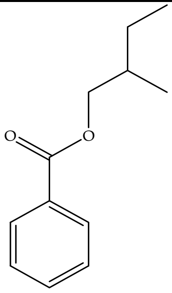	Essential oil [28]
**215**	Benzoate, isobutyl	$C_{11}H_{14}O_2$	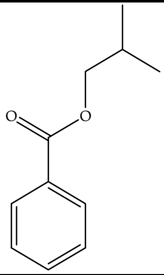	Essential oil [28]
**216**	*trans*-Chrys aenthemyl alcohol	$C_{10}H_{18}O$	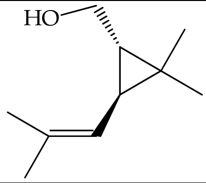	Essential oil [19]
**217**	(*E*,*E*)-2,4-Decadienal	$C_{10}H_{16}O$	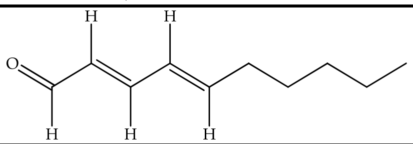	Essential oil [19, 29]
**218**	*n*-Decanal	$C_{10}H_{20}O$	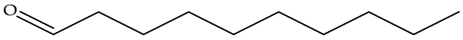	Essential oil [28]
**219**	(*E*)-2-Decenal	$C_{10}H_{18}O$	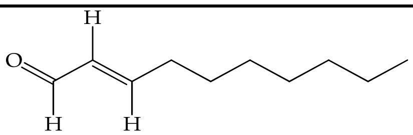	Essential oil [19]
**220**	Docosane	$C_{22}H_{46}$		Essential oil [19]
**221**	Dodecane	$C_{12}H_{26}$	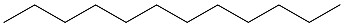	Essential oil [10]
**222**	Eicosane	$C_{20}H_{42}$		Essential oil [19]
**223**	3-Eicosyne	$C_{20}H_{38}$		Essential oil [30]

ORGANIC COMPOUNDS				
	**Compound**	**Formula**	**Structure**	**Part of *Dittrichia graveolens* L.**
			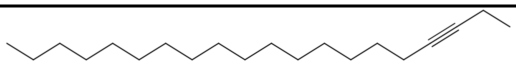	
**224**	Furfural	$C_5H_4O_2$	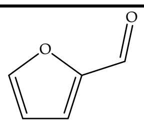	Essential oil [28]
**225**	Geranial	$C_{10}H_{16}O$	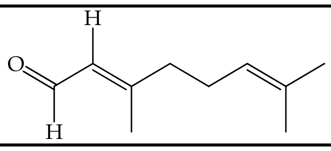	Supercritical fluid extraction (SFE) extract [25]
**226**	Heneicosane	$C_{21}H_{44}$		Essential oil [19]
**227**	(*E*,*E*)-Hepta-2,4-dienal	$C_7H_{10}O$	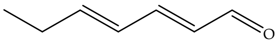	Essential oil [28]
**228**	Heptadecane	$C_{17}H_{36}$	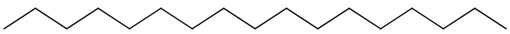	Essential oil [19, 30]
**229**	(*E*)-Hex-2-enal	$C_6H_{10}O$	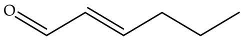	Essential oil [28]
**230**	(*Z*)-Hex-3-enyl tiglate	$C_{11}H_{18}O_2$	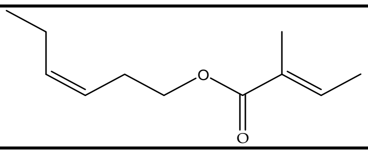	Essential oil [28]
**231**	Hexacosane	$C_{26}H_{54}$		Essential oil [19]
**232**	Hexadecane	$C_{16}H_{34}$	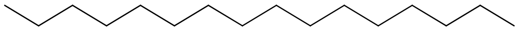	Essential oil [19]
**233**	*n*-Hexanal	$C_6H_{12}O$	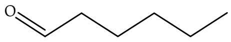	Essential oil [28]
**234**	*cis*-3-Hexenyl benzoate	$C_{13}H_{16}O_2$	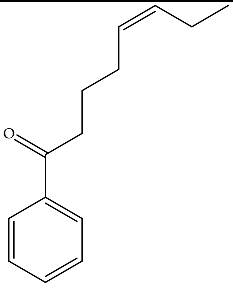	Essential oil [26]

			ORGANIC COMPOUNDS	
	**Compound**	**Formula**	**Structure**	**Part of *Dittrichia graveolens* L.**
**235**	Lavandulyl isovalerate	$C_{15}H_{26}O_2$	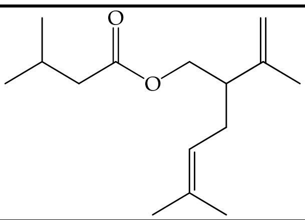	Supercritical fluid extraction (SFE) extract [25]
**236**	3-Methylbut-2-enal	C_5H_{10}	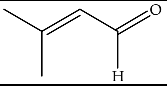	Essential oil [28]
**237**	6-Methylhept-5-en-2-one	$C_8H_{14}O$	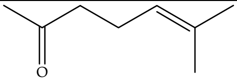	Essential oil [28]
**238**	(*E*)-Nerolidol	$C_{15}H_{26}O$	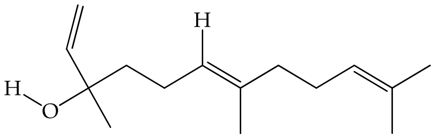	Essential oil [19, 25]<br><br>Supercritical fluid extraction (SFE) extract [25]
**239**	(*Z*)-Nerolidol acetate	$C_{16}H_{26}O_2$	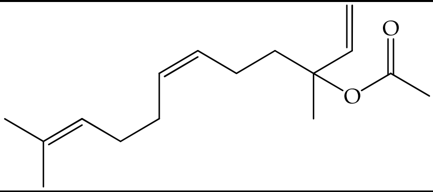	Essential oil [25]<br><br>Supercritical fluid extraction (SFE) extract [25]
**240**	Neryl acetate	$C_{12}H_{20}O_2$	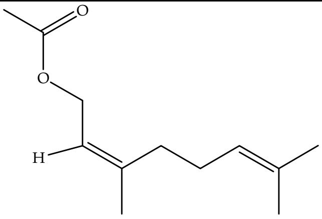	Essential oil [23]
**241**	Nonadecane	$C_{19}H_{40}$	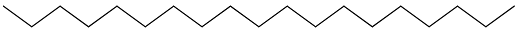	Essential oil [19]
**242**	*n*-Nonanal	$C_9H_{18}O$	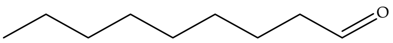	Essential oil [28, 29]
**243**	3-Nonanone	$C_9H_{18}O$	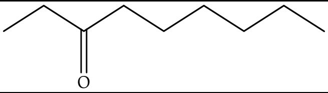	Essential oil [20]
**244**	(*E*)-*β*-Ocimene	$C_{10}H_{16}$	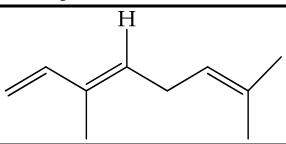	Essential oil [19]
**245**	Octadecane	$C_{18}H_{38}$	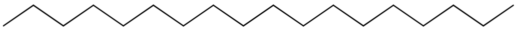	Essential oil [19]
**246**	Octanol acetate	$C_{10}H_{20}O_2$	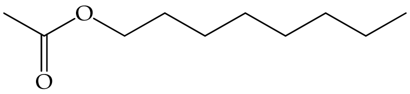	Supercritical fluid extraction (SFE) extract [25]
**247**	Pentacosane	$C_{25}H_{52}$	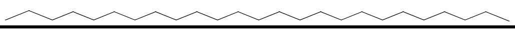	Essential oil [19]

	**ORGANIC COMPOUNDS**			
	**Compound**	**Formula**	**Structure**	**Part of *Dittrichia graveolens* L.**
**248**	Pentadecanal	$C_{15}H_{30}O$	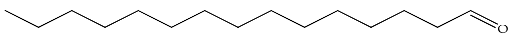	Essential oil [30]
**249**	Pentadecanone, 6,10,14-trimethyl-2	$C_{18}H_{36}O$	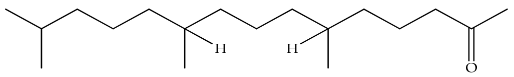	Essential oil [30]
**250**	Stearate, 2-(*p*-hydroxyphenyl)ethyl	$C_{26}H_{44}O_3$	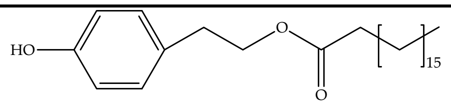	Aerial parts [17]
**251**	Tetracosane	$C_{24}H_{50}$		Essential oil [19]
**252**	Tetradecane	$C_{14}H_{30}$	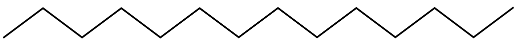	Essential oil [19, 28]
**253**	*m*-Tolualdehyde	C_8H_8O	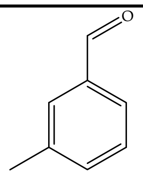	Essential oil [28]
**254**	Tricosane	$C_{23}H_{48}$		Essential oil [19]
**255**	Tridecane	$C_{13}H_{28}$	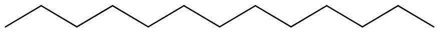	Essential oil [10, 19]
**256**	Undecane	$C_{11}H_{24}$	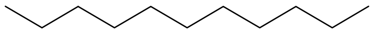	Essential oil [10]
**257**	Yomogi alcohol	$C_{10}H_{18}O$	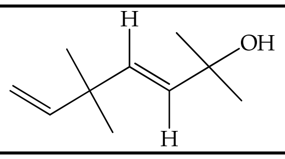	Essential oil [19]

	**OTHER COMPOUNDS**			
	**Compound**	**Formula**	**Structure**	**Part of *Dittrichia graveolens* L.**
**258**	Crypto-acorone	$C_{15}H_{24}O_2$	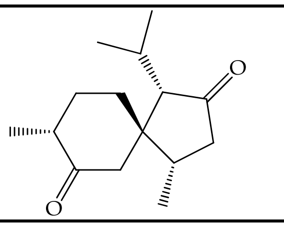	Essential oil [25]<br><br>Supercritical fluid extraction (SFE) extract [25]

OTHER COMPOUNDS				
	**Compound**	**Formula**	**Structure**	**Part of *Dittrichia graveolens* L.**
**259**	Cyclopentadecanolide	$C_{15}H_{28}O_2$	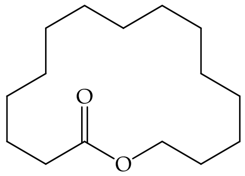	Essential oil [25]<br><br>Supercritical fluid extraction (SFE) extract [25]
**261**	8-*α*-11-Elemodiol	$C_{14}H_{24}O_2$	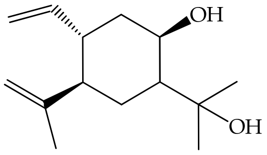	Essential oil [25]<br><br>Supercritical fluid extraction (SFE) extract [25]
**262**	8-(1-Methylethylidene)bicyclo [5.1.0] octane	$C_{11}H_{18}$	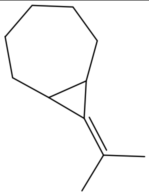	Essential oil [20]
